# Supplementary material for: Scalable synthesis of ant-nest-like bulk porous silicon for high-performance lithium-ion battery anodes
Source: Nat Commun. 2019 Mar 29;10:1447. doi: 10.1038/s41467-019-09510-5 (PMC6441089; doi:10.1038/s41467-019-09510-5)
Supplement: Supplementary file 1 — Supplementary Information [file 41467_2019_9510_MOESM1_ESM.pdf]

Supplementary Information

Scalable synthesis of ant-nest-like bulk porous silicon for high-performance lithium-ion battery anode

Weili An *et al.*

Supplementary Figures

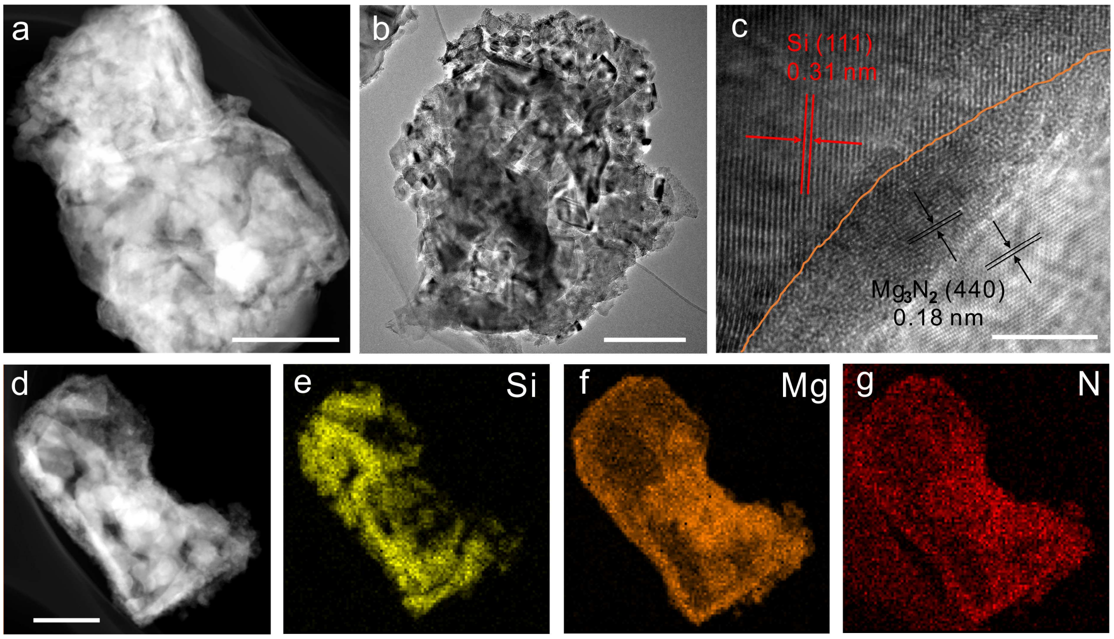

Supplementary Figure 1. Morphology characterization of Mg_3N_2/Si composites. (a) High-angle annular dark field scanning transmission electron microscopy image (scale bar = 500 nm). **(b)** TEM and **(c)** High-resolution transmission electron microscopy (HR-TEM) images of Mg_3N_2/Si (scale bar for **b** and **c** = 500 and 5 nm, respectively). **(d-g)** Energy-dispersive X-ray spectroscopy (EDS) elemental maps acquired from Mg_3N_2/Si (scale bar = 500 nm).

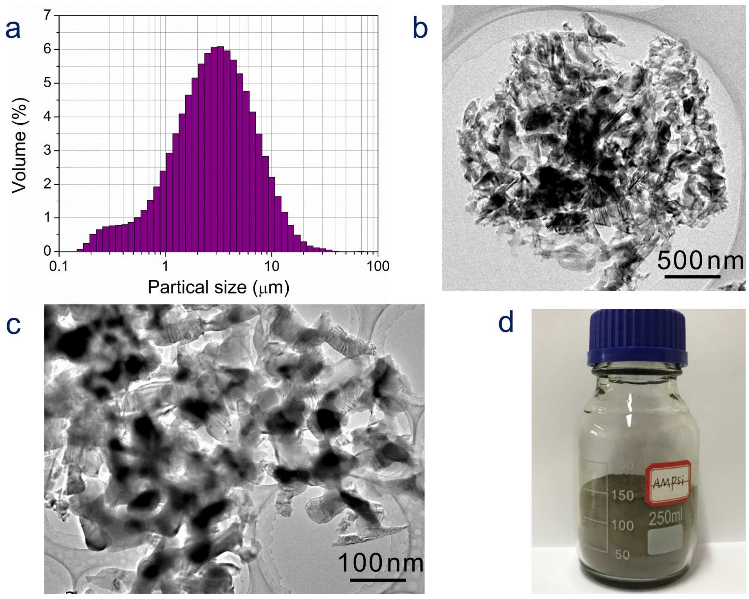

Supplementary Figure 2. Morphology and structure characterization of AMPSi. **(a)** Statistical analysis of the size distribution of AMPSi. (**b)** Low and **(c)** High magnification SEM images of AMPSi (scale bar for **b** and **c** = 500 and 100 nm, respectively). **(d)** Photograph of the final product (110 g) of AMPSi per batch synthesized in our experiments.

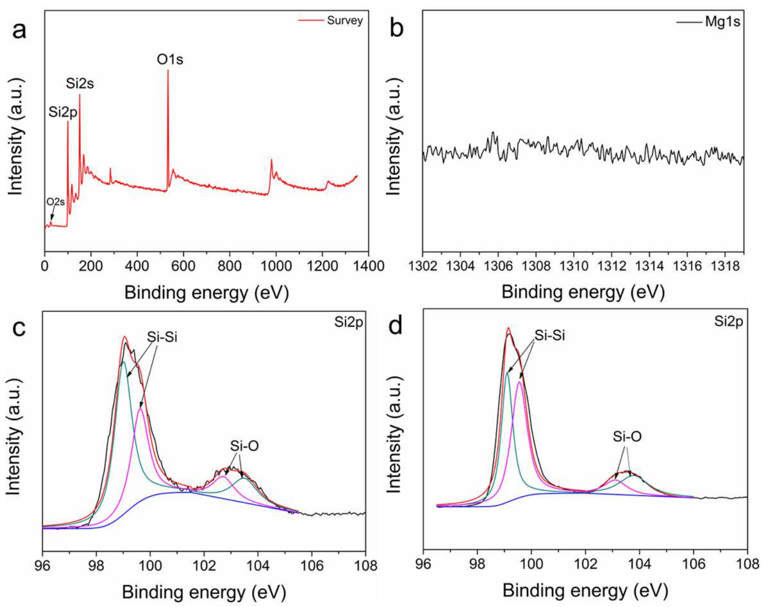

Supplementary Figure 3. XPS spectra characterization for AMPSi and AMPSi@C. XPS survey **(a)** and corresponding high-resolution spectra of Mg 1s **(b)** and Si 2p **(c)** of AMPS and high-resolution spectra of Si 2p **(d)** of AMPSi@C.

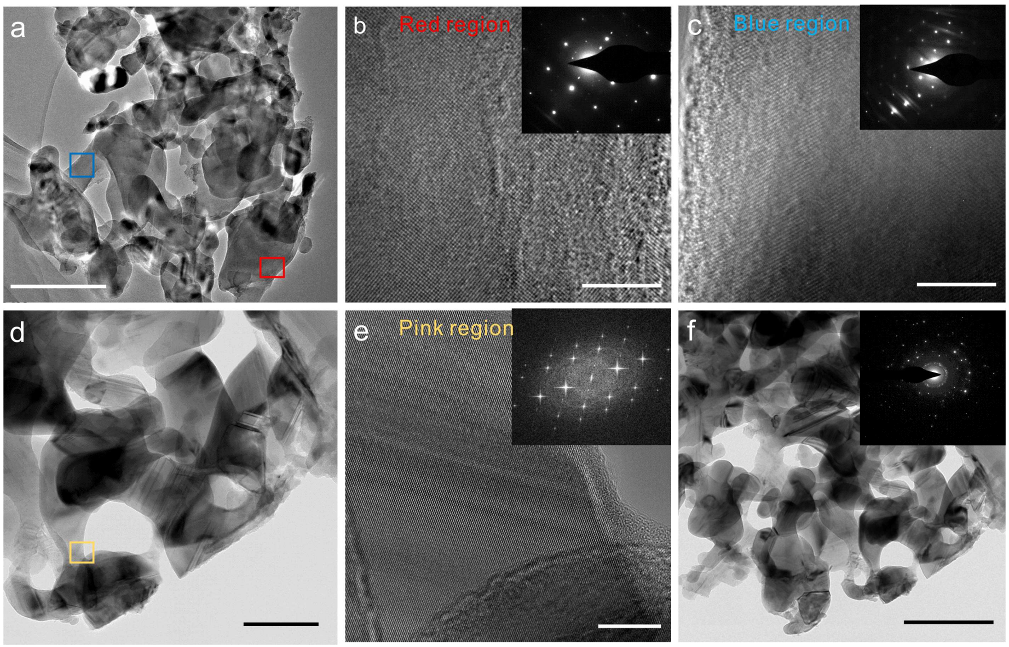

Supplementary Figure 4. Characterization of AMPSi. (a-e) HR-TEM image and corresponding selected-area electron diffraction (SEDA) patterns of AMPSi taken from different regions (scale bar for **a**, **b**, **c**, **d** and **e** = 500, 5, 5, 100 and 10 nm, respectively). **(f)** The results confirm that Si framework is a large crystalline grain of Si and AMPSi is composed of many large crystalline grains of Si (scale bar for **f** = 500 nm).

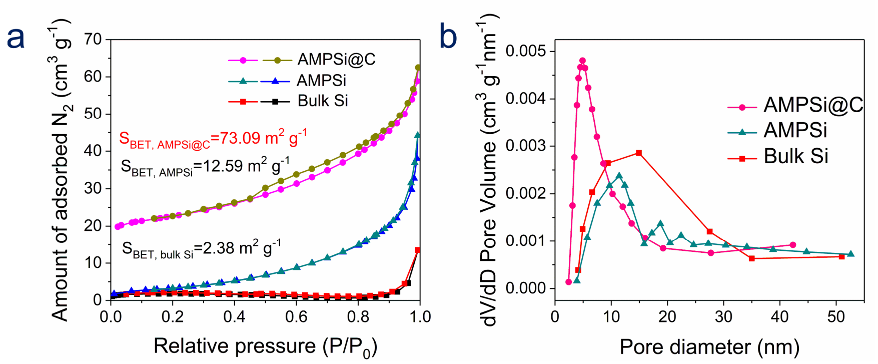

Supplementary Figure 5. Porous structure characterization of samples. (a) Nitrogen adsorption-desorption isotherms and **(b)** Corresponding pore size distribution of bulk Si, AMPSi, and AMPSi@C.

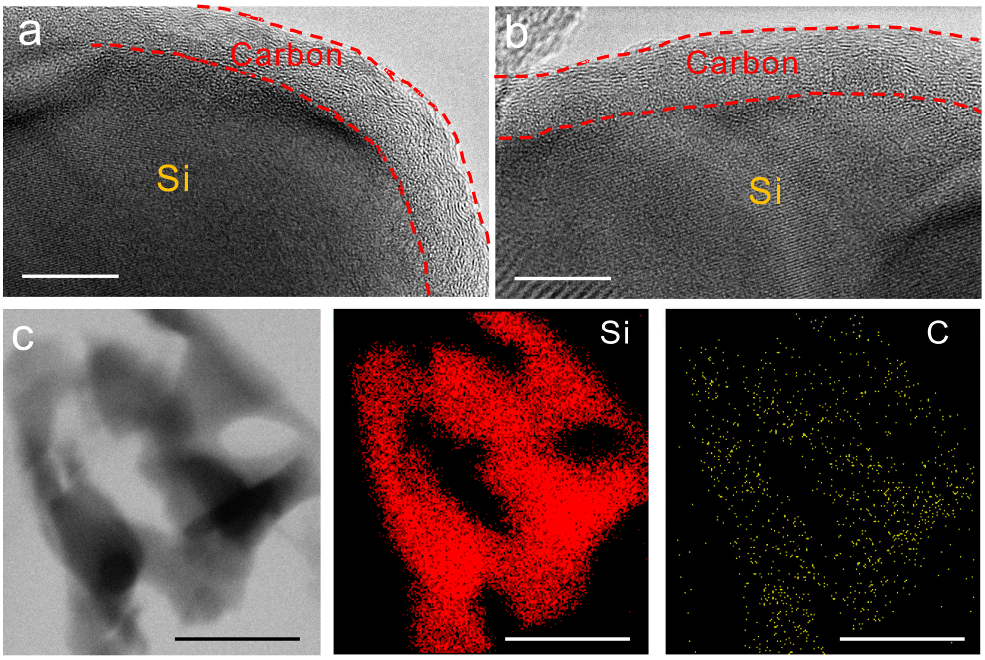

Supplementary Figure 6. Structure characterization of AMPSi@C. (a and **b)** HR-TEM image of AMPSi@C at different regions and EDS maps **(c)** of the Si frameworks in AMPSi@C with red and yellow corresponding to Si and C, respectively. Scale bar for **a**, **b** and **c** = 10, 10 and 200 nm, respectively.

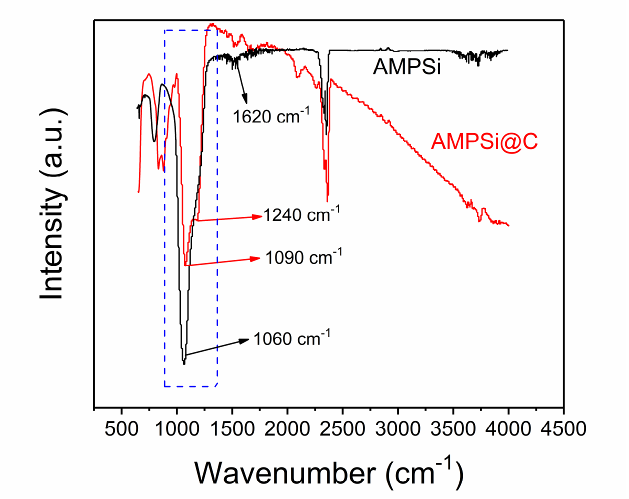

Supplementary Figure 7. FTIR spectra of the AMPSi and AMPSi@C.

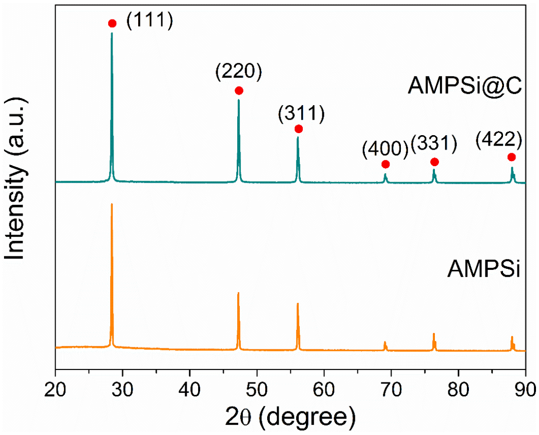

Supplementary Figure 8. XRD patterns of AMPSi and AMPSi@C.

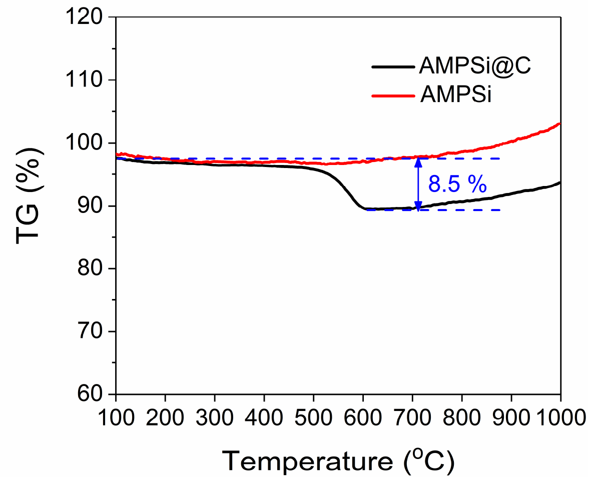

Supplementary Figure 9. Thermogravimetric analysis of AMPSi and AMPSi@C.

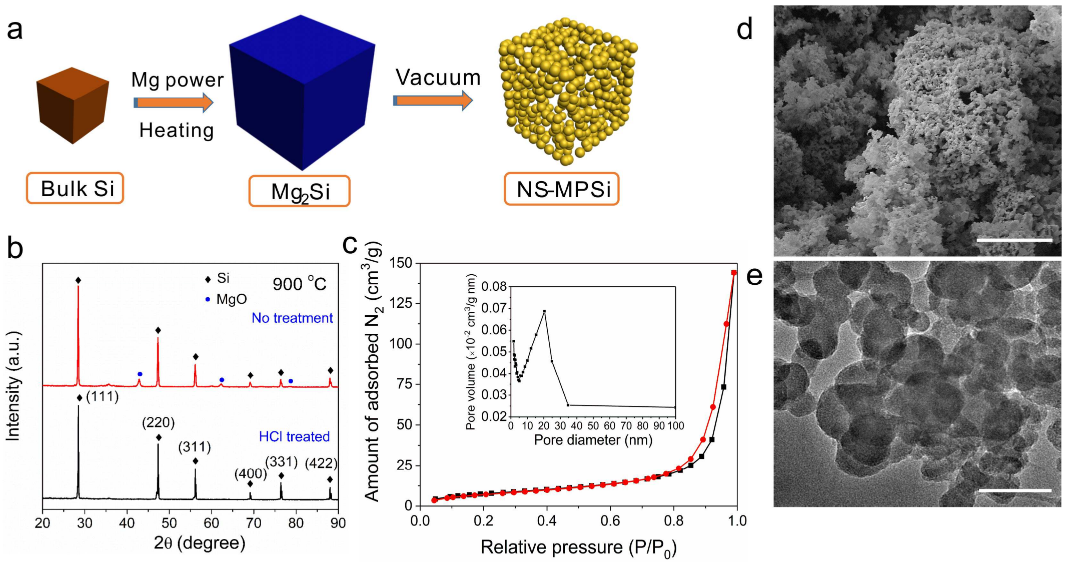

Supplementary Figure 10. Preparation and characterization of NS-MPSi. (a) Schematic illustrating the preparation procedures of NS-MPSi; **(b)** Corresponding XRD patterns and **(c)** Nitrogen adsorption-desorption isotherms; **(d)** SEM and **(e)** TEM images of NS-MPSi (Scale bar for **d** and **e** = 1 μm and 50 nm, respectively).

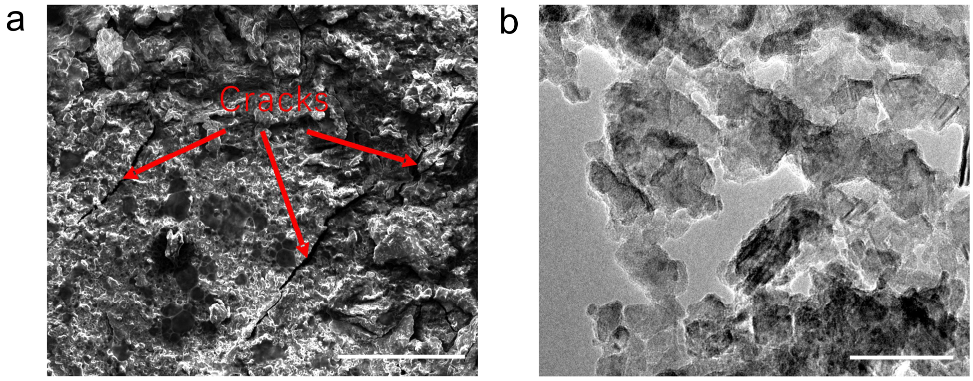

Supplementary Figure 11. Characterization of electrode film for NS-MPSi. (a) SEM image of the electrode film and HRTEM **(b)** image of NS-MPSi after cycling. Scale bar for **a** and **b** = 300 μm and 50 nm, respectively.

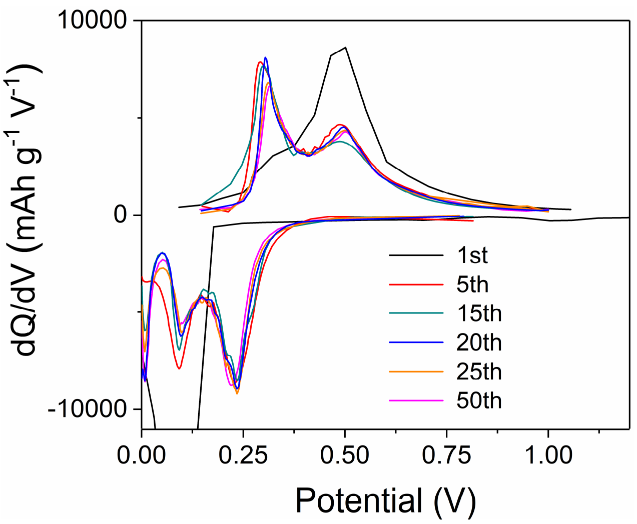

Supplementary Figure 12. Plots of differential capacity of AMPSi@C in different cycles.

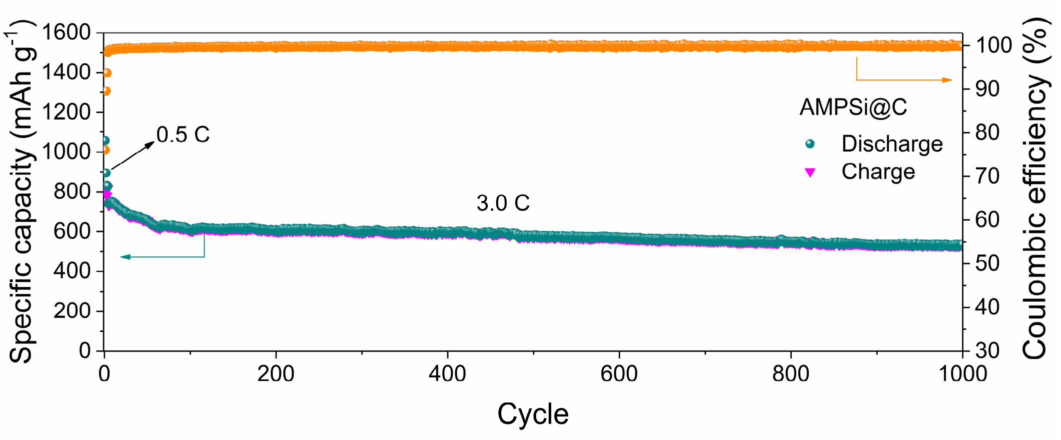

Supplementary Figure 13. Long-term cycling test conducted at 3 C for later 1,000 cycles.

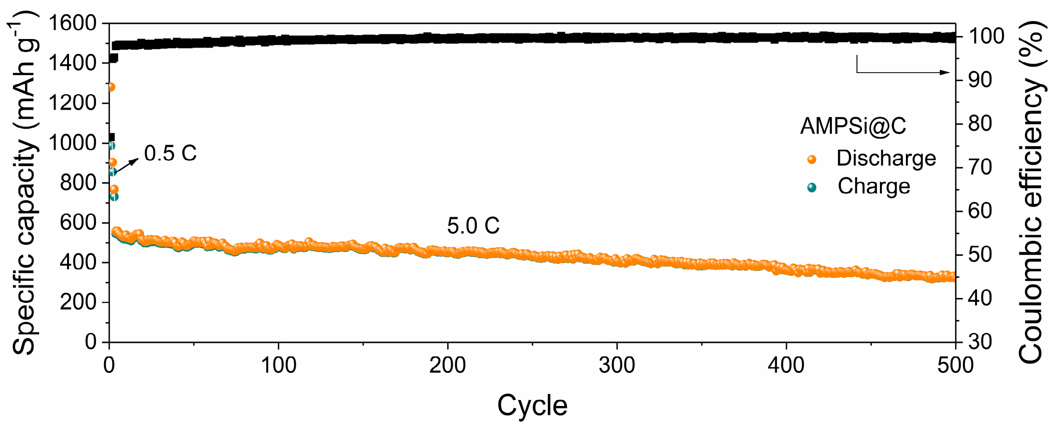

Supplementary Figure 14. Long-term cycling test conducted at 5 C for later 500 cycles (0.5 C activation for the first three cycles, 1 C = 4,200 mA g^{-1}).

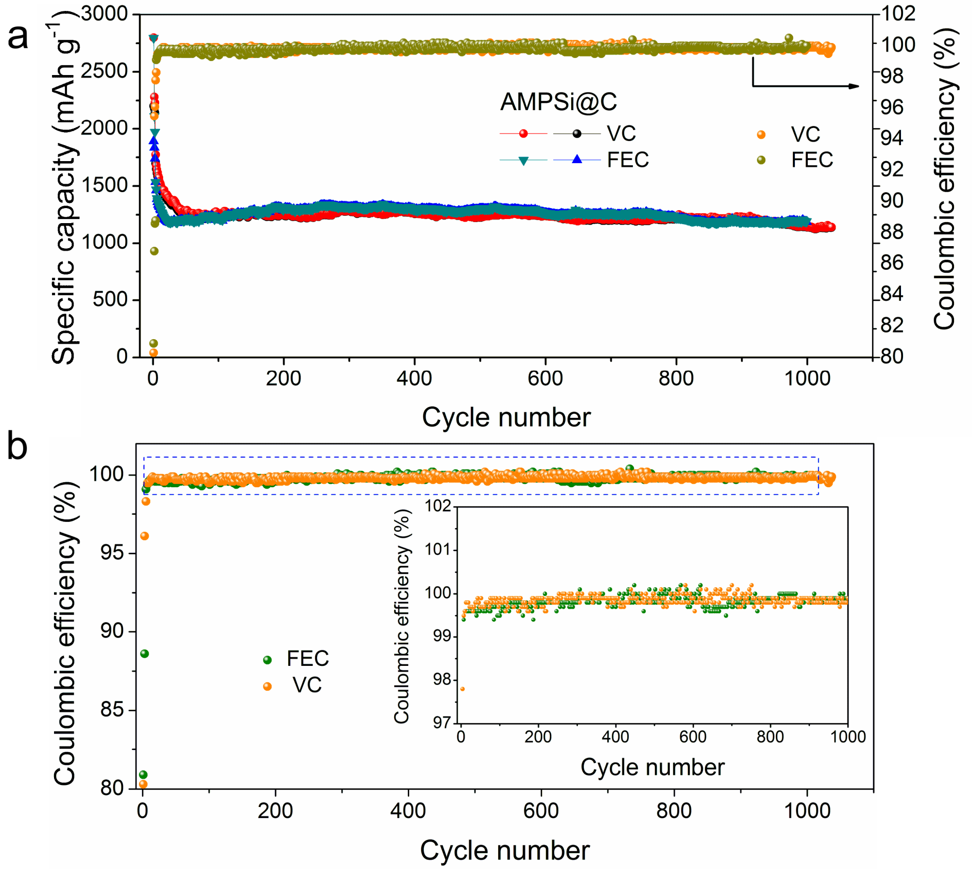

Supplementary Figure 15. **Cyclability comparison between VC and FEC.** The cycle performance **(a)** and Coulombic efficiency **(b)** of AMPSi@C electrodes in two electrolyte solutions with VC and FEC as additive

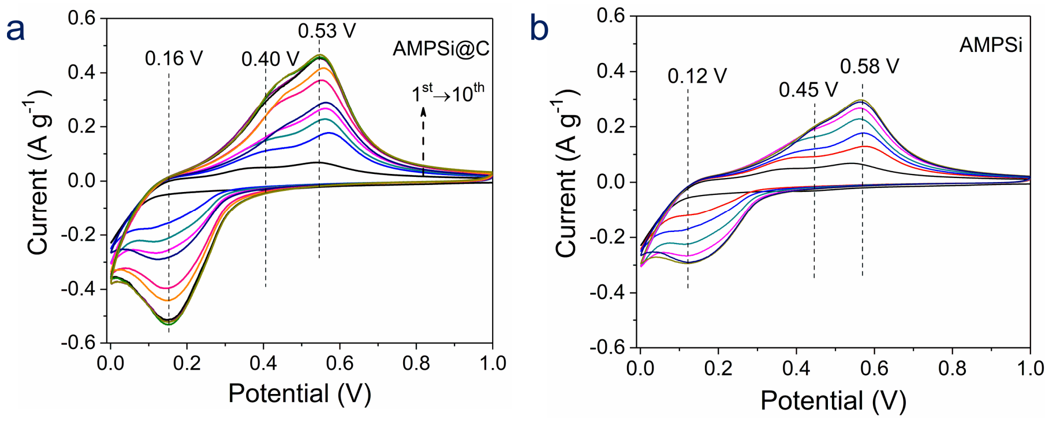

Supplementary Figure 16. Cyclic voltammograms of **(a)** AMPSi@C and **(b)** AMPSi.

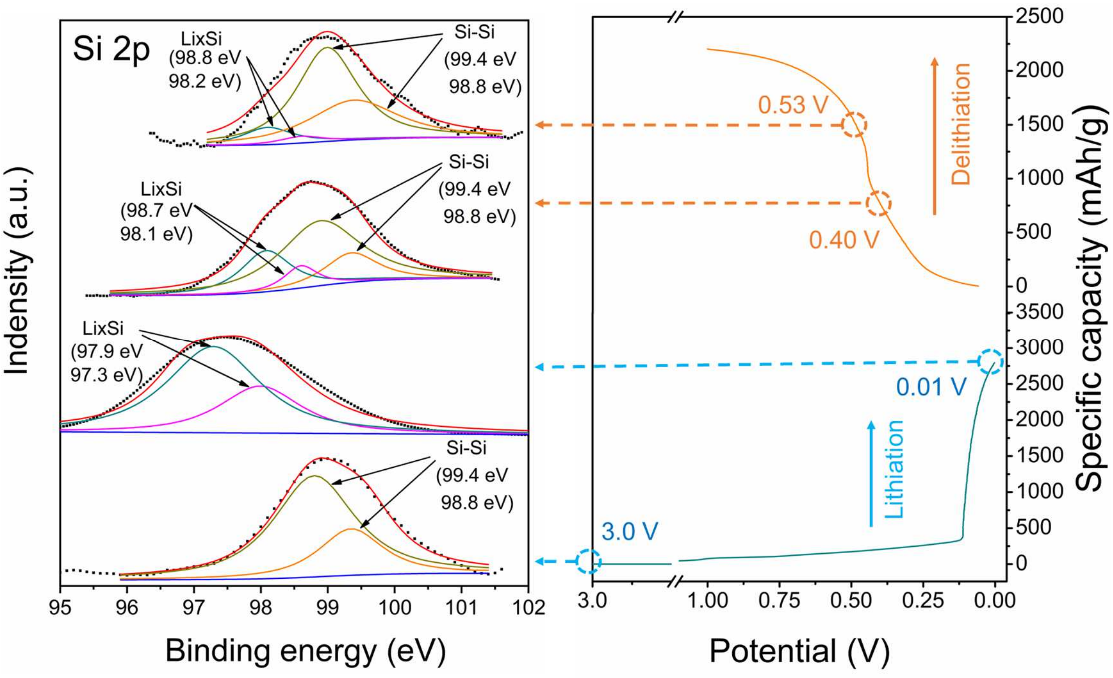

Supplementary Figure 17. Si 2p spectra of the AMPSi@C composite electrodes upon discharge/charge cycle and corresponding discharge/charge potentials. To conduct the XPS characterization, the surface solid state interlayer (SEI) on AMPSi@C was first removed by Ar^{+} ion etching.

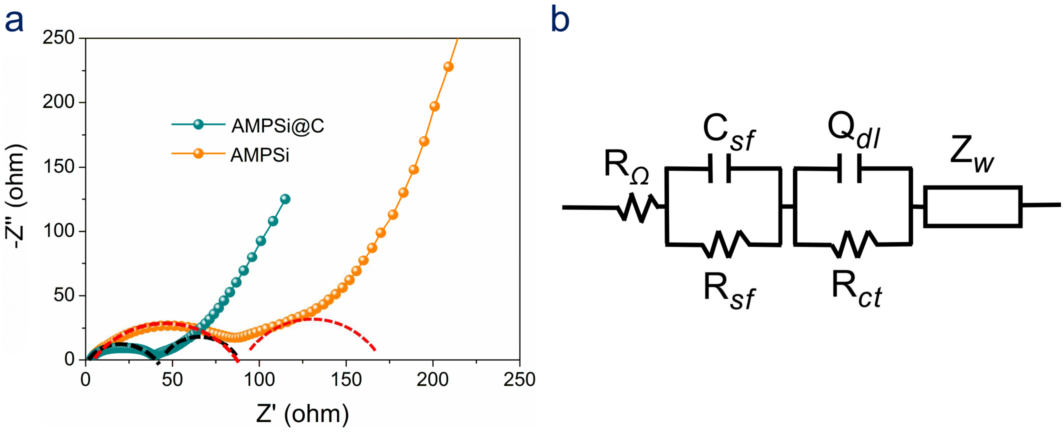

Supplementary Figure 18. Electrochemical impedance analysis of samples. (a) Electrochemical impedance spectroscopy of AMPSi and AMPSi@C and **(b)** corresponding equivalent circuit.

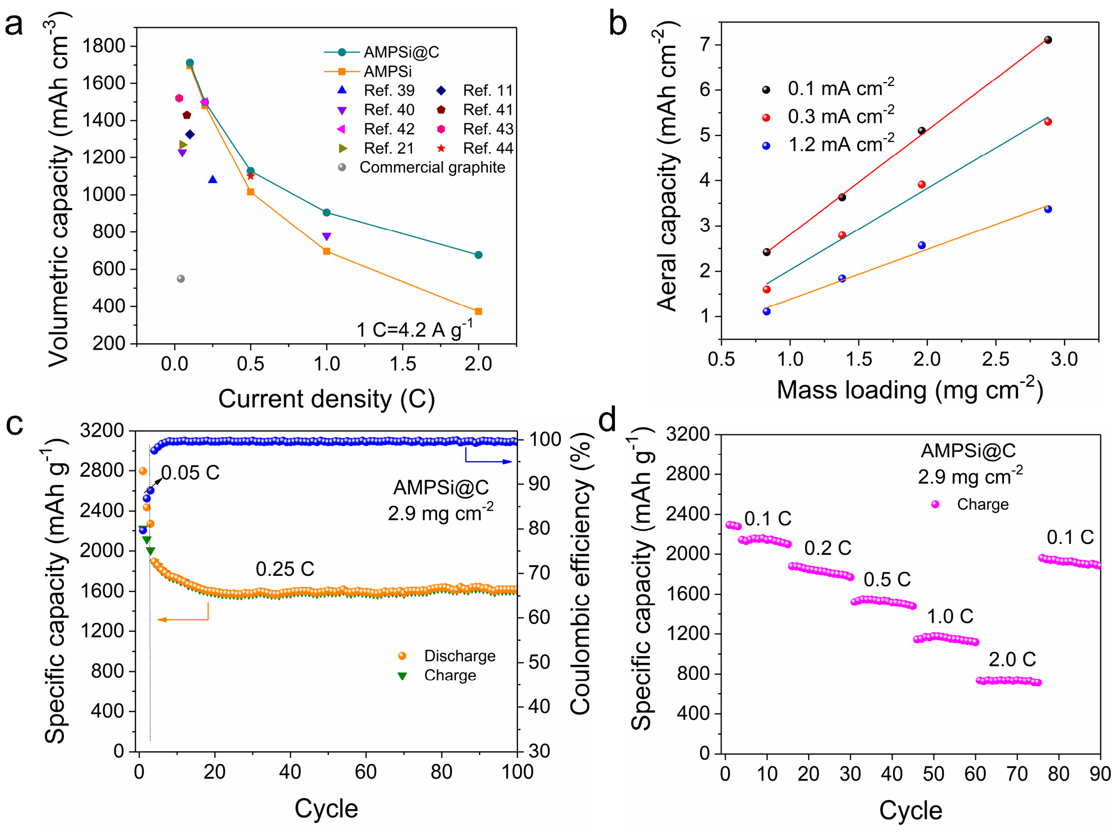

Supplementary Figure 19. Electrochemical properties of samples. (a) Volumetric capacity comparison of AMPSi, AMPSi@C, and other Si based anodes; **(b)** Dependence of the electrode areal capacity on the mass loading at different current densities with the specific capacity calculated based on the mass of AMPSi@C; **(c)** Cycling performance at 0.25 C and **(d)** Rate capacities of AMPSi@C for a large mass loading of 2.9 mg cm^{-2}.

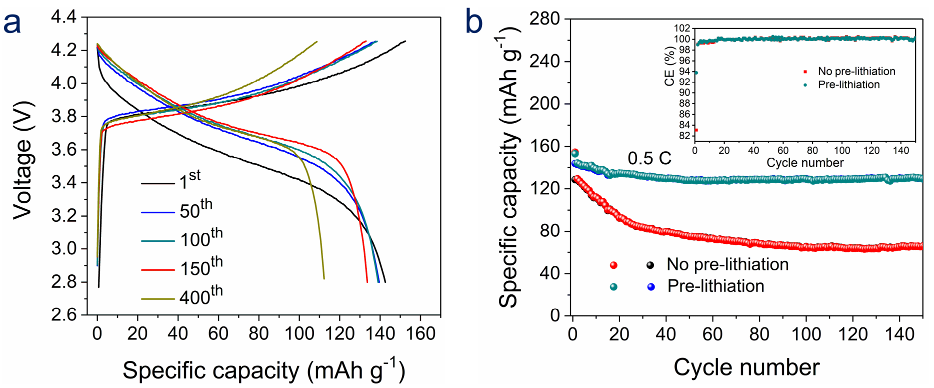

Supplementary Figure 20. (a) Charging-discharging curves of the AMPSi@C//NCM full coin after pre-activation of the AMPSi@C composite in a half cell. **(b)** Cycle performance of AMPSi@C//NCM full coin with non-prelithiated AMPSi@C as anode.

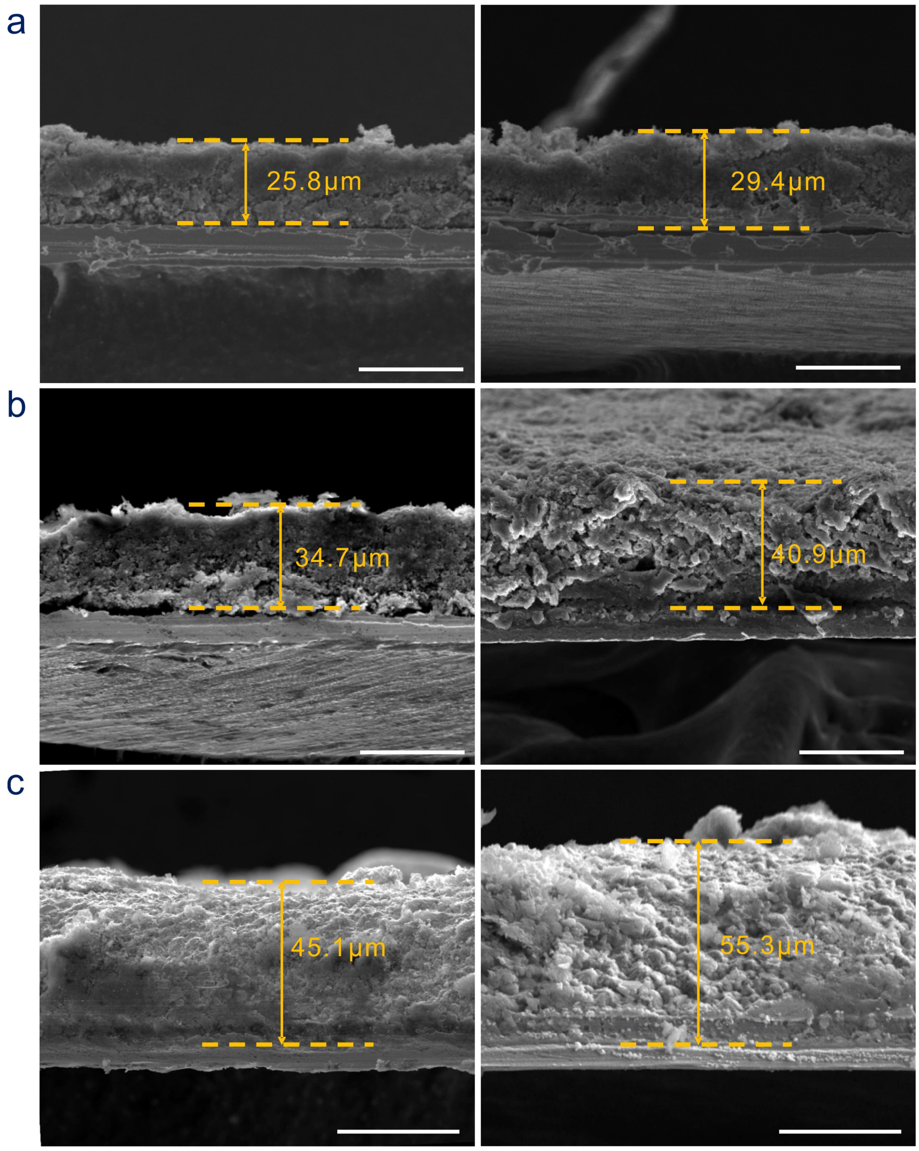

Supplementary Figure 21. Electrode swelling analysis of the AMPSi@C films. Cross-sectional SEM images acquired to investigate electrode swelling of the AMPSi@C films with different thicknesses of **(a)** 25.8, **(b)** 34.7, and **(c)** 45.1 μm together with the corresponding cross-sectional SEM images after full lithiation. Scale bar for both **a**, **b** and **c** = 30 μm.

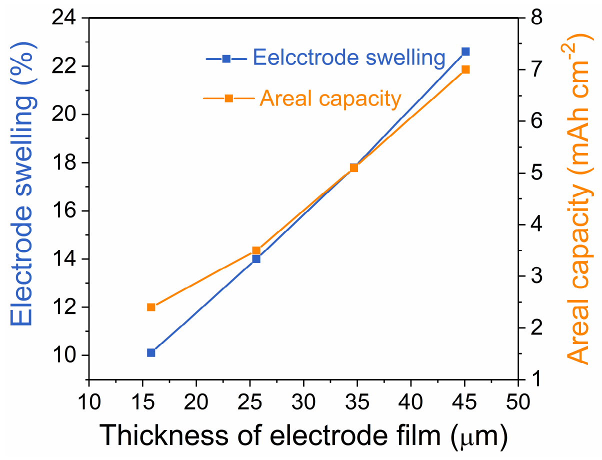

Supplementary Figure 22. Relationship between the thickness of the electrode and electrode swelling with the corresponding areal capacity of the AMPSi@C at 0.1 mA cm^{-2} plotted on the right y-axis.

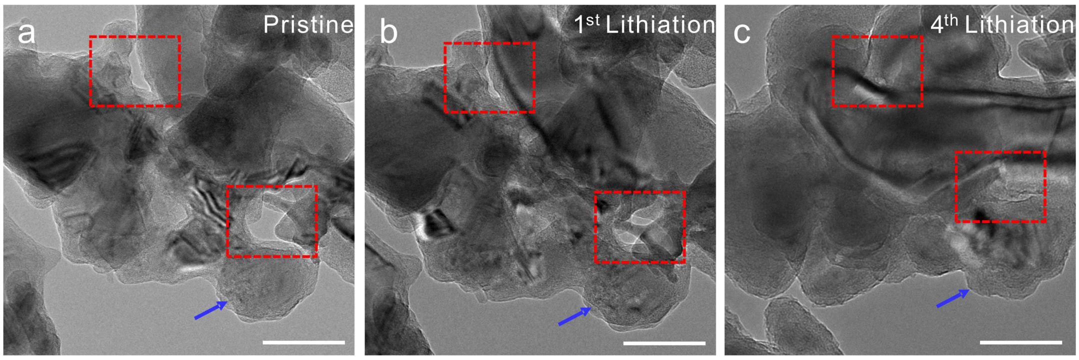

Supplementary Figure 23. *In situ* TEM characterization of of AMPSi@C. *In situ* electrochemical observation of AMPSi@C. Magnified TEM images of **(a)**, pristine, **(b)** the first lithiated, and **(c)** the fourth lithiated of AMPSi@C. Scale bar for both **a**, **b** and **c** = 100 nm.

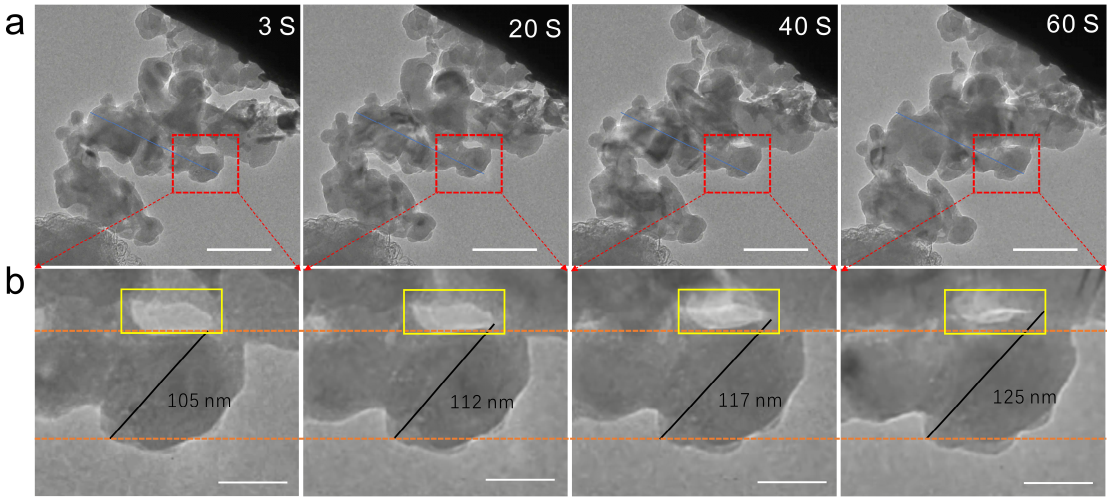

Supplementary Figure 24. ***In situ*** **electrochemical observation of AMPSi@C.** Time-resolved TEM images of AMPSi@C at different times taken from in-situ TEM test. The size of the partial Si skeleton (shown in red region) is measured to be 105, 112, 117, 125 nm after lithiation for 3, 20, 40, 60 s, respectively. Meanwhile, the pore (shown in yellow region) is filled accordingly. The display was sped up by 10 times the real time of lithiation/delithiation. Scale bar for **a** and **b** = 200 and 50 nm, respectively.

Supplementary Tables

Supplementary Table 1. Comparison of the electrochemical characteristics and other important features of AMPSi, AMPSi@C, and other reported Si/C composites.

Materials	Content of Si (%)	Tap density (g cm^{-3})	Mass loading (mg cm^{-2})	Cycling (mA h/g) based on active materials mass	Rate capacity (mAh g^{-1})	Electrode thickness swelling	ICE (%)	Capacity retention of cycles	Areal capacity (mAh cm^{-2})	Volumetric capacity (mAh cm^{-3})
This work	91.5%	0.8	0.8	1144.3 after 1000 cycles at 0.5 C	847.9 at 2 C<br>593.4 at 3 C<br>322.1 at 5 C	10.1%	80%	90%	2.8 at 0.1 mA cm^{-2}	1712 at 0.1 C
	91.5%	0.8	2.9	1614.6 after 100 cycles at 0.25 C	739.7 at 2 C	22.6%	79.6 %	88%	7.1 at 0.1 mA cm^{-2}	1760 at 0.1 C
Pomegranate Si/C (Ref.20)	77%	0.53	3.12	1,160 after 1,000 cycles at 0.5 C	690 at 2 C	-	75%	90%	3.7 at 0.03 mA cm^{-2}	1270 at 0.05 C
Micro-sized Si–C composite (Ref.11)	80%	0.78	1.2	1459 after 200 cycles at 0.24 C	990 at 1.5 C	44%	77%	97.8%	1.9 at 0.3 mA cm^{-2}	1326 at 0.1 C
Micrometer-sized porous Si (Ref.49)	82.7%	0.72	2.11	1467 after 370 cycles at 0.62 C	650 at 2.6 C	-	75%	83%	2.8 at 0.2 mA cm^{-2}	1075 at 0.06 C
Fe-Cu-Si composite (Ref.15)	~70%	0.8	8.5	420 after 50 cycles at 0.5 C	429 at 1.2 C	49%	91%	90%	3.44 at 0.18 mA cm^{-2}	1030 at 0.05 C
Porous Si sponge (Ref.26)	40%	-	0.5	570 after 1,000 cycles at 0.25 C	410 at 1 C	30%	56%	80%	1.8 at 0.06 mA cm^{-2}	-
Watermelon-Inspired Si/C Microsphere (Ref.6)	12.5 %	0.68	4.1	450 after 250 cycles at 0.5 C	~500 at 5 C	-	89.2 %	80%	2.54 at 0.2 mA cm^{-2}	~420 at 0.07 C
Si/N-doped C /CNT (Ref.14)	71%	-	0.8	1031 after 100 cycles at 0.15 C	~600 at 0.5 C	-	72%	67%	1.07 at 0.42 mA cm^{-2}	-
Si microcube (Ref.25)	81.4%	0.25	1.0	1338 after 200 cycles at 0.48 C	907 at 2.4 C	41.7%	74.7 %	77.6%	1.9 at 0.2 mA cm^{-2}	~480 at 0.05 C
Porous coral-like Si (Ref.27)	-	-	~1.0	1956 after 100 cycles at 0.01 C	971 at 2 C	65%	68.1 %	79.8%	~2.0 at 0.1 mA cm^{-2}	-
Embedded Graphitic C Shell on Si (Ref.51)	82%	-	0.9	1056 after 800 cycles at 0.48 C	1155 at 1.4 C	-	64%	~66%	1.9 at 0.2 mA cm^{-2}	-

Note： 1 C= 4.2 A g^{-1}

Supplementary Table 2. Comparison of the electrochemical performance between AMPSi@C and typical Si-based anodes reported recently.

**Anode materials**	**Cathode material**	**Full-cell capacity retention**	**Energy density (Wh kg^{-1})**
Our work AMPSi@C	$LiNi_{1/3}Co_{1/3}Mn_{1/3}O_2$	84% retention after 400 cycles	502.5
Si/C Spheres[1]	$LiCoO_2$	62.5% retention after 100 cycles	N/A
Si-C/graphene[2]	$LiNi_{0.5}Mn_{0.2}Co_{0.3}O_2$	85% retention after 100 cycles	420
Si-C/graphene[3]	$Li[Ni_{0.75}Co_{0.1}Mn_{0.15}]_2$	88.4% retention after 750 cycles	>240
Si-graphene Hybrid[4]	$LiCoO_2$	83% retention after 100 cycles	468
Si-graphene foam monolith[5]	$LiFePO_4$	91% retention after 80 cycles	N/A
Si graphene nanoribbon papers[6]	$LiCoO_2$	75% retention after 174 cycles	386
Graphene cages/Si[7]	$LiCoO_2$	90% retention after 100 cycles	N/A
Si/SiO_2[8]	$LiCoO_2$	60% retention after 100 cycles	292
$Si/LiSiO_3/Li_4Ti_5O_{12}$[9]	$LiCoO_2$	82% retention after 100 cycles	N/A
$C\text{-}SiO_x$[10]	$Li[Ni_{0.8}Co_{0.15}Al_{0.05}]O_2$	58% retention after 100 cycles	508.5
SiO[11]	$LiNi_{1/3}Mn_{1/3}Co_{1/3}O_2$	80% retention after 100 cycles	N/A
Fe–Cu–Si ternary[12]	$LiCoO_2$	63.9% retention after 300 cycles	N/A
Si-nanolayer embedded graphite[13]	$LiCoO_2$	92% retention after 100 cycles	N/A
Si/C-graphite[14]	$Li(Ni_{1/3}Mn_{1/3}Co_{1/3})O_2$	84% retention after 300 cycles	N/A

Supplementary Table 3. Comparison of the capacities based on the total mass including active material, binder and conductive additive (minus the current collector) between AMPSi@C and typical Si-based anodes reported.

**Materials**	Mass ratio of active materials, carbon black and binder (%)	Cycling capacity based on toatal materials mass (mA h g^{-1})	Rate capacity based on toatal materials mass (mAh g^{-1})
This work	80:10:10	915.4 after 1000 cycles at 0.5 C<br>1291.7 after 100 cycles at 0.25 C	678.4 at 2 C<br>474.7 at 3 C
Pomegranate Si/C (Ref.20)	80:10:10	928 after 1,000 cycles at 0.5 C	552 at 2 C
Micro-sized Si-C composite (Ref.11)	60:20:20	875.4 after 200 cycles at 0.24 C	594 at 1.5 C
Micrometer-sized porous Si (Ref.49)	70:10:20	880 after 370 cycles at 0.62 C	455 at 2.6 C
Fe-Cu-Si composite (Ref.15)	80:10:10	336 after 50 cycles at 0.5 C	343 at 1.2 C
Porous Si sponge (Ref.26)	40:40:20	228 after 1,000 cycles at 0.25 C	164 at 1 C
Watermelon-Inspired Si/C Microsphere (Ref.6)	90:5:5	405 after 250 cycles at 0.5 C	~450 at 5 C
Si/N-doped C /CNT (Ref.14)	65:20:15	670 after 100 cycles at 0.15 C	~390 at 0.5 C
Si submicrocube (Ref.25)	80:10:10	1070 after 200 cycles at 0.48 C	725.6 at 2.4 C
Porous coral-like Si (Ref.27)	70:20:10	1369 after 100 cycles at 0.01 C	680 at 2 C

Supplementary Movies

Supplementary Movie 1: *In-situ* TEM observation of lithiation of AMPSi@C composites at a constant bias of -3V (the display was sped up by 10 times the real time of lithiation).

Supplementary Movie 2: *In-situ* TEM observation of four lithiation/delithiation cycling of AMPSi@C composites (the display was sped up by 10 times the real time of lithiation/delithiation).

Supplementary Movie 3: *In-situ* TEM observation of lithiation of AMPSi@C composites by applying a bias of -3V in the preceding lithiation and suddenly increasing to -9V for the later lithiation to confirm the structural ability at a higher rate. (The display was sped up by 10 times the real time of lithiation)

Supplementary References

1. Wang, W. *et al.* Silicon and carbon nanocomposite spheres with enhanced electrochemical performance for full cell lithium ion batteries. *Sci. Rep.* **7,** 44838 (2017).
2. Zhao, X., Li, M., Chang, K. H. & Lin, Y. M. Composites of graphene and encapsulated silicon for practically viable high-performance lithium-ion batteries. *Nano Res.* **7,** 1429–1438 (2014).
3. Chae, C. *et al.* A high-energy Li-ion battery using a silicon-based anode and a nano-structured layered composite cathode. *Adv. Funct. Mater.* **24,** 3036–3042 (2014).
4. Ko, M. *et al.* Elastic a-silicon nanoparticle backboned graphene hybrid as a self-compacting anode for high-rate lithium ion batteries. *ACS Nano* **8,** 8591–8599 (2012).
5. Ma, Y. *et al.* Constraining Si particles within graphene foam monolith: Interfacial modification for high-performance Li^+ storage and flexible integrated configuration. *Adv. Funct. Mater.* **26,** 6797–6806 (2016).
6. Salvatierra, R. V. *et al.* Silicon nanowires and lithium cobalt oxide nanowires in graphene nanoribbon papers for full lithium ion battery. *Adv. Energy Mate.* **6,** 1600918 (2016).
7. Li, Y. Z. *et al.* Growth of conformal graphene cages on micrometre-sized silicon particles as stable battery anodes. *Nat. Energy* **1,** 15029 (2016).
8. Liang, J. *et al.* A deep reduction and partial oxidation strategy for fabrication of mesoporous Si anode for lithium ion batteries. *ACS Nano* **10,** 2295–2304 (2016).
9. Park, S. *et al*. High-performance silicon-based multicomponent battery anodes produced via synergistic coupling of multifunctional coating layers. *Energy Environ. Sci.* **8**, 2075–2084 (2015).
10. Kim, H. J. *et al.* Controlled prelithiation of silicon monoxide for high performance lithium-ion rechargeable full cells. *Nano Lett.* **16,** 282–288 (2016).
11. Zhao, H. *et al.* Toward practical application of functional conductive polymer binder for a high-energy lithium-ion battery design. *Nano Lett.* **14,** 6704–6710 (2014).
12. Chae, S. J. et al. Micron-sized Fe-Cu-Si ternary composite anodes for high energy Li-ion batteries. *Energy Environ. Sci*. **9**, 1251–1257 (2016).
13. Ko, M. *et al.* Scalable synthesis of silicon-nanolayer-embedded graphite for high-energy lithium-ion batteries. *Nat. Energy* **1,** 16113 (2016).

14. Li, X. L. *et al.* Design of porous Si/C-graphite electrodes with long cycle stability and controlled swelling. *Energy Environ. Sci.* **10,** 1427–1434 (2017).
