# Supplementary material for: Scalable synthesis of ant-nest-like bulk porous silicon for high-performance lithium-ion battery anodes
Source: Nat Commun. 2019 Mar 29;10:1447. doi: 10.1038/s41467-019-09510-5 (PMC6441089; doi:10.1038/s41467-019-09510-5)
Supplement: Supplementary file 3 — Description of Additional Supplementary Files [file 41467_2019_9510_MOESM3_ESM.pdf]

Description of Additional Supplementary Files

File Name: Supplementary Movie 1

Description: *In-situ* TEM observation of lithiation of AMPSi@C composites at a constant bias of -3V (the display was sped up by 10 times the real time of lithiation).

File Name: Supplementary Movie 2

Description: *In-situ* TEM observation of four lithiation/delithiation cycling of AMPSi@C composites (the display was sped up by 10 times the real time of lithiation/delithiation).

File Name: Supplementary Movie 3

Description: *In-situ* TEM observation of lithiation of AMPSi@C composites by applying a bias of -3V in the preceding lithiation and suddenly increasing to -9V for the later lithiation to confirm the structural ability at a higher rate. (The display was sped up by 10 times the real time of lithiation)
